# Experimental analysis of methylammonium and Formamidinium-based halide perovskite properties for optoelectronic applications

**DOI:** 10.1016/j.heliyon.2023.e21701

**Published:** 2023-10-28

**Authors:** Sonali Mehra, Mamta, V.N. Singh, Govind Gupta, A.K. Srivastava, Shailesh Narain Sharma

**Affiliations:** a CSIR- National Physical Laboratory, Dr. KS Krishnan Marg, New Delhi, 110012, India; b Academy of Scientific and Innovative Research (AcSIR), Ghaziabad, 201002, India; c CSIR- Advanced Materials and Processes Research Institute, Bhopal, Madhya Pradesh, India

**Keywords:** Perovskite, Non-lead, Bismuth-halide, Thickness variation, Simulation

## Abstract

Nowadays, the toxicity of lead in metal-halide perovskites is the most precarious obstruction in the commercialization of perovskite-based optoelectronic devices. However, Pb-free metal halide perovskites as environment-friendly materials because of their exceptional properties, such as band-gap tunability, narrow emission spectra, low toxicity and easy solution-processability, are potential candidates for optoelectronic applications. Recently, literature reported the poor structural stability and low-emission intensity of Bi-based perovskite NCs. Still, this paper focuses on the fabrication of Formamidinium (FA)-based Bi mixed halide and Methylammonium(MA)-based Bi-pure halide perovskites using Ligand-Assisted Reprecipitation Technique (LARP) technique. XRD diffraction patterns of FA-based perovskites were slightly broad, signifying the nanocrystalline form and limited size of perovskite nanocrystals. While the XRD diffraction patterns of MA_3_Bi_2_X_9_ (X = Cl/Br/I) perovskites were narrow, signifying the amorphous nature and larger size of perovskite nanocrystals. The peak positions were varied in MA-based bismuth halide perovskites with respect to the halide variation from Br to Cl to I ions. The optical study shows the variation in band gap and average lifetime with respect to halide variation leading to enhanced optical properties for device applications. The band-gap of FA_3_Bi_2_Br_x_Cl_1-x_ & FA_3_Bi_2_I_x_Cl_1-x_ perovskites was calculated to be around 3.7 & 3.8 eV, respectively, while in MA-halide perovskites the band-gap was calculated to be 2.8 eV, 3.1 eV & 3.4 eV with respect to halide variation from I to Cl to Br in perovskite samples using Tauc's plot respectively. Moreover, simulation is carried out using the SCAPS-1D software to study the various parameters in MA & FA-based Bi-pure or mixed halide perovskites. Here, we discussed the variation in efficiency with respect to the thickness variation from 100 to 500 nm for MA_3_Bi_2_I_9_ halide perovskites. These MA_3_Bi_2_I_9_ halide perovskites show minimum efficiency of 4.65 % at 100 nm thickness, while the perovskite sample exhibits maximum efficiency of 10.32 % at 500 nm thickness. Thus, the results stated that the thickness of absorber layers directly affects the device characteristics for optoelectronic applications.

## 1 Introduction

Hybrid organic-inorganic perovskites have materialized as prominent perovskite materials that attract the attention of researchers for energy storage, energy conversion and light-emission applications. But, still, the toxicity of lead is a pertinent issue which needs to be resolved by replacing lead (Pb) with its non-toxic substitutes to develop high light-absorbing perovskites for commercial applications. Because of this, other than Sn(II) & Ge(III), Bi(III) possess isoelectronic configuration with Pb(II) and good chemical stability [[1], [2], [3]] and thus, Bi(III) species were considered as the effective candidate for fabrication of MA & FA-based Pb-free halide perovskites. Generally, these perovskites consist of A_3_B(III)_2_X_9_ (B=Bi, Sb; X = I/Cl/Br) geometry that forms a bi-octahedron structure made of [B_2_X_9_]^3-^. Along with versatile structures, Bi-halide perovskites also show improved structural stability and captivating optoelectronic properties, such as phase transition [4], non-linear optical activity [5], and thermochromic & photochromic effect [6]. Bi-based perovskites also exhibit prominent photovoltaic device performance, and variation in halide composition helps develop colour-tunable and highly luminescent Bi-based perovskite nanocrystals. Bi-halide Pb-free perovskites were known as effective materials because of their non-toxicity, rich structural diversity and optoelectronic properties [7,8]. These Bi-halide perovskites form an ordered vacancy structure [[9], [10], [11]]. Trivalent cation (such as Bi(III) and Sb(III)) based halide perovskites were found to be a prominent candidate for Pb-free perovskite solar cells with respect to stability and lone-pair states which are favourable characteristics for photovoltaic applications [12,13].

The organic cations in A_3_B_2_X_9_ perovskite structures play a very important role in the formation of perovskite structures, and they show a large impact on the stability and optoelectronic properties of Pb-based & Pb-free halide perovskites [14,15]. Cation-substitution is important to attain the perovskite conduction band's highly stable and applicable dynamic position [16]. Thus, Methyl-ammonium (MA) and Formamidinium (FA) were known as widely studied organic cations for Pb-free organic metal-halide perovskites. However, the size and functions of the organic A-cations greatly affect the dimensionality and stability of perovskite lattice [17], whereas these MA & FA-cations also affect the physical properties, such as light absorption and charge transport in perovskite materials [18]. Here, the replacement of inorganic Cs^+^ cation with organic MA^+^ or FA^+^- ion leads to the increase in volume and band-gap of the perovskite material. Although, octahedra deformation increases with an increase in the ionic radius of organic cations [19]. Therefore, A-cation substitution was accountable for the perovskite lattice's expansion, contraction, or octahedral tilting. It will affect the band gap and the optical properties of the halide perovskites [20,21].

On the other hand, organic-inorganic bismuth halide perovskites are the major topic of discussion among researchers due to their stability in ambient atmosphere, non-toxic nature, and easy processing [22]. Generally, replacing inorganic Cs^+^ cation with organic MA^+^ & FA^+^ cation leads to enhanced photocurrent conversion and reduced band-gap for perovskite-based optoelectronic applications. These bismuth halide perovskites are stable in ambient air, but their large band gap leads to low power conversion efficiency. Moreover, Bismuth triiodide (BiI_3_) is known to be a potential light absorber due to its indirect optical band-gap and also an efficient sunlight absorber in photovoltaic applications [23]. Nowadays, researchers face the issue of utilizing BiI_3_ as an active perovskite layer for photovoltaics because of the non-aligned energy levels between bismuth halide and semiconductor [24]. It leads to the non-suitability of BiI_3_ or A_3_Bi_2_I_9_ as an absorber for perovskite lead-free perovskite solar cells [25]. However, the composite of BiX_3_ with MAX coated on a glass substrate for device fabrication leads to poor morphology, homogeneity, and low film coverage for perovskite devices [26]. Thus, all these factors hinder the photovoltaic performance of Bi-halide perovskite-based devices. Therefore, we discussed the thickness variation characteristics using the J-V curve with varying cations for the optimization of optoelectronic parameters of Bi-halide perovskites [27].

Among all Bi-based perovskite materials, FA_3_Bi_2_X_9_ perovskites were air-stable and environment-friendly [22,28]. These FA-based FA_3_Bi_2_X_9_ perovskites have achieved photoconversion efficiency comparable to Pb-based perovskite materials, and these Pb-free perovskites with increased band-gap lead to higher recombination for optoelectronic applications. However, A-site cations such as Cs^+^, MA^+^ and FA^+^ affect the band-gap of Pb-based perovskites, such as perovskites with large radius A-site cations (FAPbI_3_) possess lower band-gap and better photovoltaic performance in comparison with small radius A-site cations (MAPbI_3_, CsPbI_3_) based perovskite materials [[29], [30], [31]]. On the contrary, FA-based bismuth perovskites have rarely been reported, and thus, their characteristics and optoelectronic performance remain unknown. As per the literature, photovoltaic conversion efficiencies (PCEs) reported for Cs_3_Bi_2_I_9_ and MA_3_Bi_2_I_9_ are 1.09 % and 0.12 % [28], which can be improved to 3.2 % & 1.64 %, respectively by recrystallizing [12] and increasing the grain boundary [32] size of perovskite thus enhancing the optical properties of Pb-free perovskite materials.

Thus, FA & MA-based Bi-halide perovskites exhibit higher band-gap with good optical properties for optoelectronic applications. Therefore, this work discusses the synthesis and characterization of FA & MA-based bismuth halide perovskites- FA_3_Bi_2_Br_x_Cl_1-x_ & FA_3_Bi_2_I_x_Cl_1-x_ and MA_3_Bi_2_X_9_ (X = Cl/Br/I) using Ligand-Assisted Reprecipitation (LARP) technique and studied their properties for potential optoelectronic applications. Thus, dual-mixing in FA-based perovskites to stabilize its structure has rarely been explored, and optimization of dual-mixing of halide composition is prominent for developing non-toxic FA-based Pb-free optoelectronic devices. Similarly, MA-based Bi-pure halide perovskites have been studied widely for MA_3_Bi_2_I_9_. In contrast, Cl^−^ & Br^−^ halide anions-based perovskites are very less explored and studied their optoelectronic properties with respect to the variation in cation & anion composition.

## 2 Experimentation & characterization

### 2.1 Experimental technique

**Materials:** All the chemicals required for synthesis, such as Formamidinium Bromide (CH_5_BrN_2_, ≥98 %), Formamidinium Iodide (CH_5_IN_2_, ≥99.99 % anhydrous), Bismuth Chloride (BiCl_3_, 99.99 % trace metals basis), Methylammonium bromide (MABr, >99 % anhydrous), Methylammonium chloride (MACl, >99 %), Methylammonium iodide (MAI, >99 % anhydrous), Oleyl-amine (OLA, Tech.70 %), Oleic-acid (OA, 90 %), Octylamine (OCA, 99 %), Toluene (C_6_H_5_CH_3_, 99.8 % anhydrous), Octane (≥99 %, anhydrous), Ethyl acetate (EA, ≥99.7 %), Ethanol (99.8 %), and N, N-Dimethylformamide (DMF, 99.8 % anhydrous) were purchased from Sigma Aldrich. All the chemicals were used without any further purification.

**Fig. 1:**
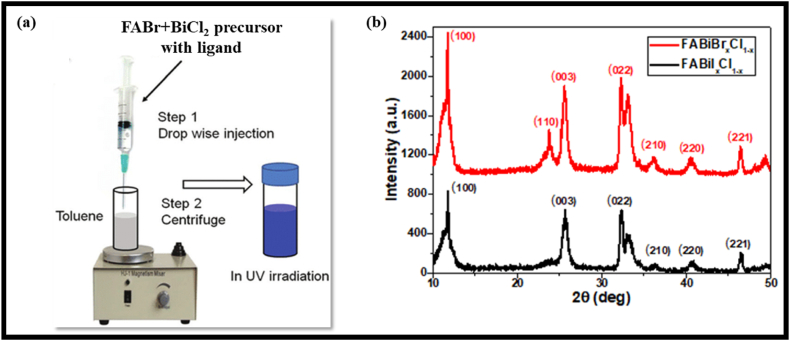
(a) Diagrammatic representation of LARP technique, and X-ray Diffraction patterns of (b) FA_3_Bi_2_Br_x_Cl_1-x_, & FA_3_Bi_2_I_x_Cl_1-x_.

In this paper, the FA & MA-based bismuth halide perovskites were synthesized using the Ligand-Assisted Reprecipitation technique (LARP) and a diagrammatic representation of this technique is presented in Fig. 1(a). The LARP technique is simple and versatile for synthesizing organic-inorganic Pb-free perovskite QDs and NCs [33,34]. Reprecipitation through solvent mixing is a simple way to simultaneously prepare organic nanoparticles or polymer quantum dots. Hybrid perovskite NCs are soluble in many polar solvents but are insoluble in non-polar ones. Based on the polar and non-polar solvents, this method fabricates perovskite NCs via solvent mixing with the assistance of long-chain organic capping ligands. In the synthesis of FA-based halide perovskites, FAX and BiX_3_ precursor salts were dissolved in polar solvents DMF and ethyl acetate, respectively.

Further, the mixture of these solutions was added dropwise to the poor non-polar solvent toluene at the reaction temperature of 60–70 °C, and this solubility difference induces the formation of perovskite material where toluene as poor solvent helps in the precipitation of perovskite NCs. Here, octylamine was added to control the growth rate of perovskite NCs, while OA played an important role in stabilizing the colloidal solution of perovskite NCs during the synthesis process.

On the other hand, in the synthesis of MA-based halide perovskites, MABr and BiBr_3_ precursor salts were dissolved in polar solvents DMF and ethyl acetate, respectively, and OLA as capping ligand was added to the precursor solution. Further, these precursor solutions were added dropwise to the poor non-polar solvent octane with OA at a reaction temperature of 60–70 °C. This solubility difference induces the formation of the perovskite material. Further, these solutions were cooled down and centrifuged to obtain the supernatant for MA_3_Bi_2_Br_9_ perovskite NCs. Secondly, the halide exchange process was carried out to synthesize MA_3_Bi_2_X_9_ (X = Cl/I) perovskite NCs. Here, MACl and MAI precursor salts were dissolved in ethanol and added to the finally obtained supernatant of MA_3_Bi_2_Br_9_ perovskite solution in a stochiometric ratio. Then, these solutions were stirred for an optimum time at an optimized temperature to obtain MA_3_Bi_2_X_9_ (X = Cl/I) perovskite colloidal solutions. These MA-based pure halide perovskites were utilized for characterization.

Moreover, this synthesis process ensures the enhanced optical properties and uniform size distribution of perovskite NCs. The investigation of solvent-precursor interactions would help provide insight into the influence of solvents on the formation process and morphology-controlled perovskite QDs. This as-synthesized MA & FA-based bismuth halide perovskite film was utilized for XRD (10-80°), UV–Vis (200–800 nm), Photoluminescence, FE-SEM & TEM characterizations to optimize the device efficiency with respect to thickness variation.

However, the perovskite nanocrystals are usually ionic, while the surrounding of capping ligands around these perovskites during synthesis makes them more ionic and labile [35]. Even the addition of polar solvents into the perovskite solution during purification leads to the loss of emission property and colloidal stability of perovskite nanocrystals because of the dynamic nature between nanocrystals and ligand bonding. But this issue can be resolved by modifying the bonding equilibrium between NCs and ligands because it is very important to stabilize the addition of an excess of octylamine & OA ligands during the purification. These excess ligands form an ionic atmosphere around the perovskite NCs surface, improving colloidal stability and enhanced optical properties [35]. Here, OA plays an important role in stabilizing the colloidal solution of perovskite NCs during the synthesis process. Also, these perovskite NCs with capping ligands were more stable and showed luminescence properties for a sufficient time, denoting the optimum utilization of Bi halide perovskites for optoelectronic applications. Further, XRD characterization in the range of 10-80° has been performed to study the structural properties of the perovskites.

Here, Table 1 given below shows the designation of samples with the major synthesis conditions of all MA & FA-based bismuth halide perovskites.

**Table 1:** Designation of Samples with respective Synthesis Condition Parameters.

S.No.	Sample Name	Synthesis Method/Temperature	Solvent/Capping Ligand	Band-gap (eV)	Decay time (ns)
1.	MA_3_Bi_2_Br_9_	Ligand Assisted Reprecipitation (LARP)/60–70 °C	DMF + Ethyl acetate/Octane + Oleic acid	3.4	5.4
2.	MA_3_Bi_2_Cl_9_	LARP/60–70 °C	Ethanol/Octane + Oleic acid	3.1	4.9
3.	MA_3_Bi_2_I_9_	LARP/60–70 °C	Ethanol/Octane + Oleic acid	2.8	4.8
4.	FA_3_Bi_2_Br_x_Cl_1-x_	LARP/60–70 °C	DMF + Ethyl acetate/Toluene + Oleic acid + Oleyl-amine	3.7	1.72
5.	FA_3_Bi_2_I_x_Cl_1-x_	LARP/60–70 °C	DMF + Ethyl acetate/Toluene + Oleic acid + Oleyl-amine	3.8	2.99

Table 2 shows the comparison of previously fabricated FA & MA-based Bismuth pure halide perovskite devices and their efficiency for solar-cell applications. On the other hand, this article shows the synthesis of FA & MA-based Bismuth pure & mixed halide perovskites synthesized using the Hot-injection Method and studies their properties via numerical simulation. Here, we have synthesized MA_3_Bi_2_X_9_ (X = Cl/Br/I) and FA_3_Bi_2_Br_x_Cl_1-x_ & FA_3_Bi_2_I_x_Cl_1-x_ perovskites that have been very less explored to date. However, we have evaluated the efficiency of MA_3_Bi_2_I_9_ perovskite with respect to thickness variation using SCAPS simulation, but due to the non-availability of literature and some basic parameters, this study is not possible for other halide perovskites. Although, we are working to determine the required values theoretically shortly to study their simulation for solar-cell applications. Therefore, the study of synthesis technique and structural & optical properties of FA & MA-based halide perovskites are less explored and be an interesting research topic for young researchers.

**Table 2:** Comparison of Previously Fabricated FA & MA-Bismuth halide Perovskite Devices.

S.No.	Device Architecture	Synthesis Method	V_OC_ (V)	J_SC_ (mA/cm^2^)	FF	PCE (%)	Reference
1.	FTO/TiO_2_/meso-TiO_2_/(FA)_3_Bi_2_I_9_/spiro-MeOTAD/Au	Spin-coating	0.45	0.11	0.459	0.022	[36]
2.	FTO/meso-TiO_2_/(MA)_3_Bi_2_I_9_/spiro-MeOTAD/Au	Chemical Vapor Deposition	0.40	0.11	0.36	0.016	[37]
3.	FTO/TiO2/(MA)_3_Bi_2_I_9_/Spiro-OMeTAD/Au	Numerical Simulation with thickness variation	1.37	8.24	88.09	9.94 (@200 nm)	[38]
4.	FTO/TiO_2_/meso-TiO_2_/(MA)_3_Bi_2_I_9_/P3HT/Au	Spin-coating	0.35	1.16	0.46	0.19	[39]
5.	ITO/TiO_2_/meso-TiO_2_/(MA)_3_Bi_2_I_9_/spiro-MeOTAD/MoO_3_/Ag	Spin-coating	0.67	1.00	0.62	0.42	[40]
6.	FTO/TiO_2_/meso-TiO_2_/(FA)Na_0.25_Bi_0.25_Pb_0.5_I_3_/Au	Spin-coating	0.35	4.23	0.35	0.52	[41]

### 2.2 Characterization

The X-ray diffraction (XRD) patterns of perovskite samples were recorded using Rigaku ultima-IV X-ray Diffractometer. The diffraction pattern was recorded using CuKα wavelength (λ = 1.54056 Å) at a step rate of 0.02^o^min^−1^. PL & TRPL spectra were recorded using the Edinburgh instrument, model FLS F980, at different excitation and emission wavelength for FA-based Pb-free perovskite samples. As-prepared and degraded perovskite films absorbance maxima and band gap were recorded using UV–Visible spectroscopy; model ocean optics F300 in the 200–800 nm range.

## 3 Results and discussions

### 3.1 X-ray diffraction study

Here, Fig. 1 (b) demonstrates the XRD pattern of FA-based Pb-free mixed halide perovskite (FA_3_Bi_2_Br_x_Cl_1-x_ & FA_3_Bi_2_I_x_Cl_1-x_) film grown on glass substrates. The significant reflections at 11.78, 23.73, 25.62, 32.28, 40.61, and 46.46° 2Ѳ positions correspond to (100), (110), (003), (022), (220), and (221) planes of FA_3_Bi_2_Br_x_Cl_1-x_ perovskite, while the reflections at 2Ѳ positions of 11.84, 25.53, 32.33, 40.68, and 46.51° correspond to (100), (003), (022), (220), and (221) planes of FA_3_Bi_2_I_x_Cl_1-x_ perovskite. The peak positions were varied in FA-based Pb-free halides with respect to the halide variation in both the perovskites. This difference in peak positions was observed because of the dominance of Br^−^ and I^−^ ions in FA_3_Bi_2_Br_x_Cl_1-x_ and FA_3_Bi_2_I_x_Cl_1-x_ perovskites, respectively. XRD diffraction patterns of FA_3_Bi_2_Br_x_Cl_1-x_ & FA_3_Bi_2_I_x_Cl_1-x_ perovskites were slightly broad, signifying the nanocrystalline form and limited size of perovskite nanocrystals and the orientation of these XRD peaks in specific direction denotes the formation of layered Bi-perovskite structure [34]. However, some extra peaks were also observed in the XRD patterns, which may appear due to some impurity phases in the perovskite structure. Therefore, it was revealed that both the perovskite samples show major reflections at similar 2θ positions except for the variation in halide composition, thus, exhibiting crystallographic orientation at specific planes. Here, FWHM values for FA_3_Bi_2_Br_x_Cl_1-x_ & FA_3_Bi_2_I_x_Cl_1-x_ perovskites were calculated numerically [42,43] using origin software and shown in Table .3.

**Table 3:** FWHM values analysed numerically for FA-based Perovskites (FA_3_Bi_2_I_x_Cl_1-x_ & FA_3_Bi_2_Br_x_Cl_1-x_).

Sample Name	FWHM values at planes					
	(100)	(110)	(003)	(022)	(220)	(221)
**FA**_**3**_**Bi**_**2**_**I**_**x**_**Cl**_**1-x**_	1.5783	–	0.9043	1.6132	0.6616	0.3624
**FA**_**3**_**Bi**_**2**_**Br**_**x**_**Cl**_**1-x**_	1.2898	1.5302	0.7268	1.5135	0.7463	0.3754

**Fig. 2:**
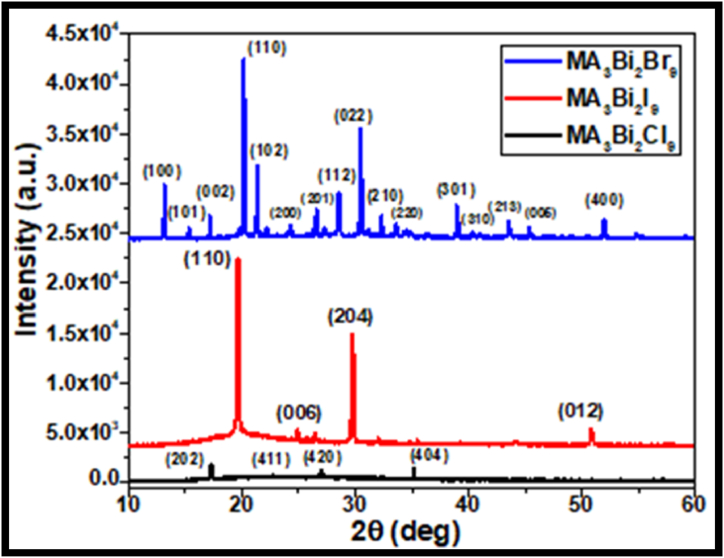
X-ray Diffraction patterns of MA_3_Bi_2_X_9_ (X = Cl/Br/I) halide perovskites.

Fig. 2 demonstrates the XRD pattern of MA-based bismuth-free halide perovskites MA_3_Bi_2_X_9_ (X = Cl/Br/I) film grown on glass substrates. The major significant reflections at 13.23, 20.19, 21.41, 28.56, 30.47, 39.02, and 52.01° 2Ѳ positions correspond to (100), (110), (102), (112), (022), (301) and (400) planes of MA_3_Bi_2_Br_9_ perovskite. The colloidal perovskites MA_3_Bi_2_X_9_ (X = Cl/I) were synthesized using the MA_3_Bi_2_Br_9_ parent solution and deposited on glass substrates to study the crystalline phase study using XRD. It was observed that the reflections at 2Ѳ positions of 19.69, 24.95, 29.77 and 50.80° correspond to (110), (006), (204), and (012) planes of MA_3_Bi_2_I_9_ perovskite and it confirms that the halide ion has been exchanged to I^−^ anion with no impurities [44]. Similarly, the reflections at 2Ѳ positions of 17.35, 22.79, 26.98 and 35.15° correspond to (202), (411), (420), and (404) planes of MA_3_Bi_2_I_9_ perovskite. The peak positions were varied in MA-based bismuth halide perovskites with respect to the halide variation from Br to Cl to I ions. XRD diffraction patterns of MA_3_Bi_2_X_9_ (X = Cl/Br/I) perovskites were narrow, signifying the amorphous nature and larger size of perovskite nanocrystals. However, some extra peaks were also observed in the XRD patterns, which may appear due to some impurity phases or capping ligands in the perovskite structure. However, the pure form of MA_3_Bi_2_Br_9_ perovskite shows a higher number of peaks. In the case of other halide perovskites, repeated washing and purification leads to the refined XRD structure of MA_3_Bi_2_X_9_ (X = Cl/I) perovskites. Therefore, all the bismuth-halide perovskites with respect to halide variation exhibit crystallographic orientation at specific planes. FWHM values for MA_3_Bi_2_X_9_ (X = I/Cl/Br) perovskites were calculated numerically using origin software and shown in Table 4a, Table 4b, Table 4c(a to c).

**Table 4a:** FWHM values analysed numerically for MA_3_Bi_2_I_9_.

Sample Name	FWHM values at planes			
	(110)	(006)	(204)	(012)
MA3Bi2I9	0.1772	9.4425	0.1862	0.2488

**Table 4b:** FWHM values analysed numerically for MA_3_Bi_2_Cl_9_.

Sample Name	FWHM values at planes			
	(202)	(411)	(420)	(404)
**MA**_**3**_**Bi**_**2**_**Cl**_**9**_	0.1289	9.2651	15.9312	54.6946

**Table 4c:** FWHM values analysed numerically for MA_3_Bi_2_Br_9_.

Sample Name	FWHM values at planes								
	(100)	(101)	(002)	(110)	(102)	(200)	(201)	(112)	(022)
**MA**_**3**_**Bi**_**2**_**Br**_**9**_	0.2099	0.0016	0.2551	0.0918	0.1166	0.2749	0.5151	0.2012	0.1362
**Sample Name**	**FWHM values at planes**								
	**(210)**	**(113)**	**(023)**	**(301)**	**(213)**	**(005)**	**(400)**	**(402)**	**-**
**MA**_**3**_**Bi**_**2**_**Br**_**9**_	0.1877	0.2266	0.5824	0.1549	0.2088	0.2084	0.2155	0.1781	–

### 3.2 Optical properties

#### 3.2.1 UV–visible study

**Fig. 3:**
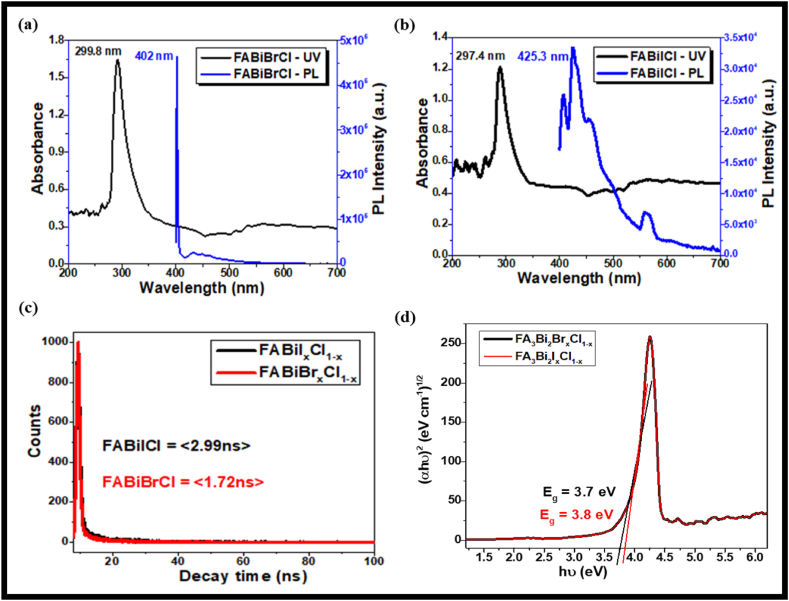
UV & PL spectra of (a) FA_3_Bi_2_Br_x_Cl_1-x_, (b) FA_3_Bi_2_I_x_Cl_1-x_, (c) TRPL spectra of FA_3_Bi_2_Br_x_Cl_1-x_, and FA_3_Bi_2_I_x_Cl_1-x_, (d) Tauc's plot of FA_3_Bi_2_Br_x_Cl_1-x_, and FA_3_Bi_2_I_x_Cl_1-x_.

The optical properties of FA_3_Bi_2_Br_x_Cl_1-x_ & FA_3_Bi_2_I_x_Cl_1-x_ perovskite nanocrystals in toluene solution were characterized using UV–Vis and PL spectroscopy as shown in Fig. 3 (a & b). The UV–Vis spectra of FA_3_Bi_2_Br_x_Cl_1-x_ & FA_3_Bi_2_I_x_Cl_1-x_ perovskite nanocrystals show a narrow, sharp absorption peak at 299.8 & 297.4 nm, respectively, that arises due to the strong electron-hole coupling interaction in lower-dimensional perovskite-like crystal structures [45,46]. Although, the absorption spectra of FA-based Bi-halide perovskite nanocrystals are much sharper compared to the previously reported Cs and MA counterparts. It is due to the larger size of FA-cation in comparison with MA or Cs-cation that provides extra space in between the Bi-halide octahedra layers creating the higher electron-hole coupling energy [47]. The band-gap of FA_3_Bi_2_Br_x_Cl_1-x_ & FA_3_Bi_2_I_x_Cl_1-x_ perovskites was calculated to be around 3.7 & 3.8 eV, respectively, using Tauc's plot (Fig. 3(d)), which agrees well with the experimentally obtained indirect band-gap for the perovskite samples. Here, the FA_3_Bi_2_Br_x_Cl_1-x_ perovskite nanocrystals exhibit higher absorption maxima leading to lower band-gap in comparison with lower absorption maxima for FA_3_Bi_2_I_x_Cl_1-x_ with higher band-gap or vice-versa. In UV–vis spectra of FA-based Bi-mixed halide perovskites, by controlling anion exchange reactions, the band-gap can be varied with a change in halide ion from Br to Cl to I leading to composition-dependent luminescence. Thus, FA_3_Bi_2_Br_x_Cl_1-x_ and FA_3_Bi_2_I_x_Cl_1-x_ perovskite exhibits band-gap of 3.7 eV & 3.8 eV respectively. Generally, it was observed that in the valence band region, the 2p orbitals of C & N atoms overlap with the 1s orbitals of H-atom, constituting the FA-cations.

In contrast, the valence-band states were majorly dominated by the halide ions. Moreover, below the fermi-level, 2p orbitals of the N-atom get hybridized with the p-orbitals of halide ions representing that the interaction between the X^−^ ion and N causes the formation of a valence band maximum. Whereas, above the fermi-level, there was the hybridization between 6p orbitals of Bi and p-orbitals of halide ions to form the conduction band minimum, which represents its position at higher energy in comparison to the valence band maximum [47].

**Fig. 4:**
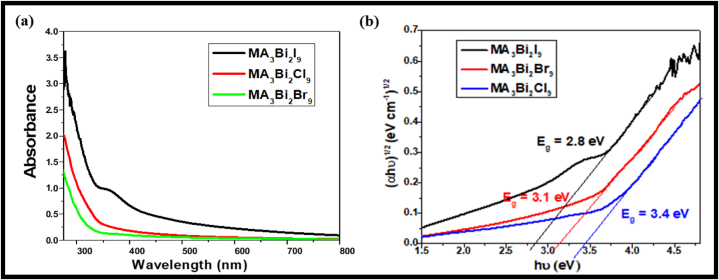
(a) UV–Visible spectra and (b) Band-gap calculation using Tauc's plot for MA_3_Bi_2_Br_9_, MA_3_Bi_2_Cl_9_, and MA_3_Bi_2_I_9_ perovskites.

The optical properties of MA-based bismuth halide perovskite nanocrystals in octane solution were characterized using UV–Vis and PL spectroscopy, as shown in Fig. 4(a). The UV–Vis absorption spectra of MA_3_Bi_2_X_9_ (X = Cl/Br/I) perovskite nanocrystals were recorded in the 200–800 nm range. The absorption edge of MA_3_Bi_2_X_9_ halide perovskites lies in the UV–visible region, and thus, these materials were also used widely as absorbers in optoelectronic devices. In the optical absorption process, a neighbouring band-edge electron is excitedly showing the transition from VB to CB via excitonic transition or band-to-band transition with photon-matter interaction. Here, in indirect transitions, the interaction between photons and lattice vibrations takes place in the electron-photon exchange process, and thus, the optical band gap is affected by the microstructural crystallite defects. Here, the optical band-gap energy of the halide perovskites was calculated using Tauc's Plot [15,48]. The band gap of the material is calculated using equation (1):

(1) (αhυ) ^n^ = A (hυ − Ε_g_)

where, α **=** Absorption coefficient.

E_g_ = Band-gap of the material
A = Constant
hυ = Energy of the incident photon (eV)
n = nature of transition (n = 2 & ½ for direct & indirect band-gap respectively)

However, direct and indirect band-gap differs based on the emission of a photon. In the present case of indirect band-gap, the photon was passed through the intermediate state and did not emit directly and also transfer the momentum of a crystal lattice [49,50].

Although, the absorption coefficient (α) can be calculated using Beer's Lambert law according to equation (2):

(2) I = I_o_ e^−αt^, (α = 2.303 * A/t)

Where A = Absorbance of material.

t = Thickness of the cuvette used in the analysis (1 cm)

Further, the graph was plotted between hυ on the x-axis and (αhυ)^2^ on the y-axis, and extrapolating the graph to obtain the band-gap of the halide perovskite samples [51,52] as shown in Fig. 4(b).

Moreover, as the halogen ion composition varies from Br^−^ to Cl^−^ to I^−^ in MA_3_Bi_2_X_9_ perovskites, the absorption band-edge shifts towards the higher wavelength and low band-gap region. Therefore, the optical properties of perovskite material were affected with respect to the variation in halide composition. Although, the results also inferred that the optical band gap varies with the different sizes of halide ions. Further, the iodide ion has the most significant size with the lowest band gap. Other halide perovskites with Br^−^ & Cl^−^ ion possesses small atomic sizes, leading to increased optical band-gap; even extrapolation creates large iodine cavities for organic molecules. Fig. 4 shows the UV–Visible spectra and the band-gap calculation of the MA_3_Bi_2_X_9_ perovskite sample [53]. Therefore, MA_3_Bi_2_X_9_ halide perovskites exhibit a band gap of 2.8 eV, 3.1eV & 3.4 eV corresponding to I^−^, Cl^−^ & Br^−^ halides respectively.

Generally, the band gap and size of the nanoparticles are inversely related. Thus, the band gap increases with the reduced particle size or vice-versa due to the proximity between the electron-hole pair, which enhances their interaction and leads to higher energy. Therefore, the high band-gap value depicts that high energy will be required to excite an electron from the valence band to the conduction band leading to a higher frequency and low wavelength or blue shift in the absorption spectra. Thus, it can be concluded that higher band-gap or blue shift in absorption spectra in halide perovskite samples has promising applications in sensors, solar cells and photodetectors [47,54].

#### 3.2.2 Photoluminescence study

Photoluminescence (PL) measurements were measured using a PL spectrophotometer in the 300–800 nm range. These FA-based Bi-halide perovskites show sharp PL spectra in the visible region under the excitation range of 370 nm. Here, FA_3_Bi_2_Br_x_Cl_1-x_ perovskites exhibit higher PL intensity as compared to FA_3_Bi_2_I_x_Cl_1-x_ perovskites. The FA_3_Bi_2_I_x_Cl_1-x_ perovskites show sharp PL emission at 425.3 nm, whereas the FA_3_Bi_2_Br_x_Cl_1-x_ perovskites also show narrow PL emission in the range of 402 nm, as shown in Fig. 3(a and b). With halide variation from I to Br with Cl^−^ ion, redshift PL emission was observed, leading to low-trap density in the materials.

Moreover, the PL emission peak was observed at different excitation wavelengths corresponding to FA_3_Bi_2_Br_x_Cl_1-x_ & FA_3_Bi_2_I_x_Cl_1-x,_ and this difference in excitation spectra was attributed to the inorganic part of the perovskite structure. However, the variation in PL emission was observed with halide variation due to structural distortion in compounds, and PL peaks suggest an independent behaviour from halide ion orbital mixing [55]. The PLQY of perovskite NCs in toluene solution was measured using the reference dye Rhodamine 6G, and the PLQY was calculated to be 4.22 % & 0.416 % corresponding to FA_3_Bi_2_Br_x_Cl_1-x_ & FA_3_Bi_2_I_x_Cl_1-x_ respectively.

Time-resolved photoluminescence (TRPL) measurements were done using the 266 nm laser for excitation. It helps to determine the decay in perovskites concerning exposure time. Here, we have also calculated the average carrier lifetime using the given formula in equation (3) for decay time [[56], [57], [58]]:

(3) $$\tau_{av}=\frac{a_{1}\;\tau_{1}^{2}+a_{2}\;\tau_{2}^{2}}{a_{1}\tau_{1}+a_{2}\;\tau_{2}}$$

Where, a_i_ = Amplitude of the ith lifetime component.

τ_i_ = Respective lifetime value

a_i_ & τ_i_ are fitting parameters described based on the fit results of the perovskite material. Using the above formula, the carrier lifetime was calculated to be 1.72 ns and 2.99 ns for FA_3_Bi_2_Br_x_Cl_1-x_ & FA_3_Bi_2_I_x_Cl_1-x_ perovskites, respectively as shown in TRPL spectra in Fig. 3(c). Hence, the decay lifetime follows the order: FA_3_Bi_2_I_x_Cl_1-x_ (2.99 ns) > FA_3_Bi_2_Br_x_Cl_1-x_ (1.72 ns). Among these FA-based Bi-halide perovskites, FA_3_Bi_2_I_x_Cl_1-x_ exhibits a higher average lifetime than FA_3_Bi_2_Br_x_Cl_1-x_ perovskites, leading to higher charge separation in I-based perovskites than Br-based perovskite nanocrystals but the difference in lifetime is not much higher to discriminate them for various applications. Thus, it can be inferred that both FA_3_Bi_2_I_x_Cl_1-x_ and FA_3_Bi_2_Br_x_Cl_1-x_ halide perovskites can be utilized for photovoltaic applications. However, the FA-based Bi halide perovskites show a sharp PL emission spectrum signifying the purity of perovskite material for display technologies and thus, due to little self-absorption among these NCs, FA_3_Bi_2_I_x_Cl_1-x_ and FA_3_Bi_2_Br_x_Cl_1-x_ perovskites can be utilized for lighting applications.

**Fig. 5:**
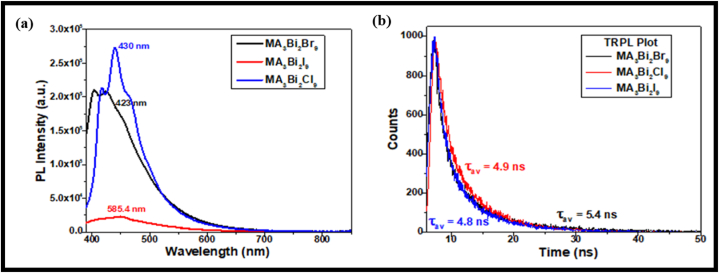
(a) Photoluminescence and (b) TRPL spectra for MA_3_Bi_2_Br_9_, MA_3_Bi_2_Cl_9_, and MA_3_Bi_2_I_9_ perovskites.

Photoluminescence (PL) measurements were measured using a PL spectrophotometer in the 400–800 nm range. The bismuth halide perovskite shows a broad band of PL emission at room temperature. In all MA_3_Bi_2_X_9_ (X = Cl, Br, I) perovskites, strong and broad PL emission was observed in the 400–800 nm range. PL spectra of MA_3_Bi_2_I_9_ perovskite show broad PL emission at 585 nm while narrow PL emission peak was observed at 423 nm and 430 nm corresponding to MA_3_Bi_2_Br_9_ and MA_3_Bi_2_Cl_9_ perovskites, respectively, as shown in Fig. 5(a). However, it was unusually observed that there was no overlap between absorption and PL emission. This weak self-absorption property benefits their utilization as phosphors in lighting applications. As the halide composition varies from I to Cl to Br, blue shift PL emission with a narrower PL peak was observed, leading to low trap density in the materials.

Moreover, the PL emission peak was observed at different excitation wavelengths for all the bismuth-halide perovskites, and this difference in excitation spectra was attributed to the presence of other energy states in the material due to non-uniform grain size distribution in all the perovskites. However, the variation in PL emission was observed with halide variation due to structural distortion in compounds, and PL peaks suggest an independent behaviour from halide ion orbital mixing [59]. It was also observed that the optoelectronic properties of bismuth halide perovskites directly depend on the (BX_3_) part in perovskites, and MA^+^ cations have no direct role in optoelectronic applications.

Time-resolved photoluminescence (TRPL) measurements were done using the 266 nm laser for excitation. It helps to determine the decay in perovskites concerning exposure time. Here, we have also calculated the average carrier lifetime using equation (3) for decay time.

Using the formula given in equation (3), the carrier lifetime was calculated to be 4.8 ns, 4.9 ns and 5.4 ns for MA_3_Bi_2_I_9_, MA_3_Bi_2_Cl_9_ and MA_3_Bi_2_Br_9_ perovskite, respectively, as shown in TRPL spectra in Fig. 5(b). Hence, the decay lifetime follows the order: MA_3_Bi_2_Br_9_ (5.4 ns) > MA_3_Bi_2_Cl_9_ (4.9 ns) > MA_3_Bi_2_I_9_ (4.8 ns). Among these MA-based Bi-halide perovskites, MA_3_Bi_2_Br_9_ perovskite exhibits a higher average lifetime compared to other bismuth-halide perovskites, leading to higher charge separation and predominant exciton radiative recombination [60] in Br-based perovskites than Cl & I^−^ based perovskite nanocrystals. Thus, amongst all MA-based bismuth-halide perovskites, MA_3_Bi_2_Br_9_ were known as potential candidates for optoelectronic applications. However, the MA-based Bi halide perovskites show a sharp PL emission spectrum signifying the purity of perovskite material for display technologies. Thus, due to self-absorption among these halide perovskites, they can be well-utilized for lighting applications.

## 4 Study of optoelectronic properties via thickness variation

**Fig. 6:**
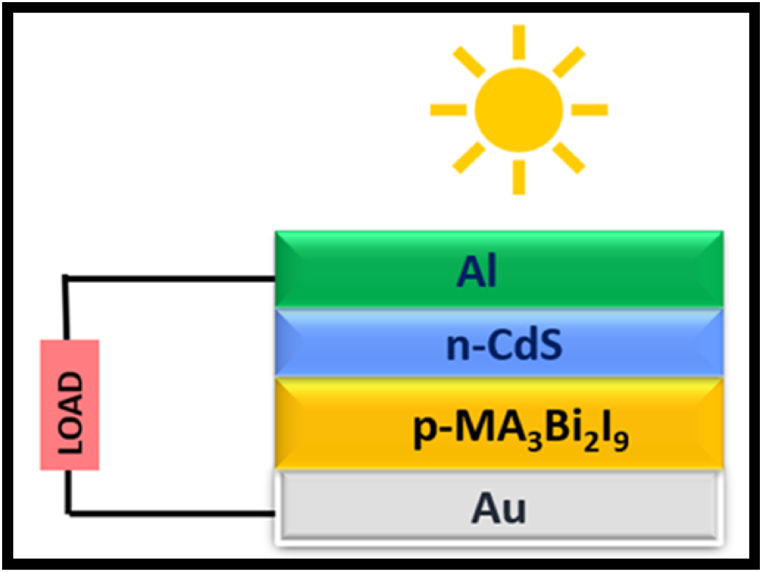
Schematic of MA_3_Bi_2_I_9_ absorber-based solar structure.

After synthesizing methylammonium Pb-free halide perovskites and characterizing their structural and optical properties using XRD, UV–Vis, PL and TRPL, the P–V potential is studied through SCAPS-1D simulation [61]. Fig. 6 shows the schematic of Optoelectronic parameters obtained through experimental investigations utilized to perform the simulation study along with the other input parameters from the literature [62,63]. However, as per the author's knowledge literature reports the simulation parameters of MA_3_Bi_2_I_9_ halide perovskites [64,65] (refer to Table 5 for input parameters) while the input parameters of simulation for MA_3_Bi_2_X_9_ (X = Cl/Br) and FA-based mixed halide perovskites is not reported in the literature. Due to this, this paper reports the simulation study of the MA_3_Bi_2_I_9_ perovskite while the study of other MA & FA-based halide perovskite is still in progress for diverse applications.

**Table 5:** Photovoltaic parameters for various layers of the device structure.

Parameters		CdS	MA_3_Bi_2_I_9_
Thickness (μm)		0.05	Varying (0.1–0.5)
Band Gap (eV)		2.40	2.2
Electron Affinity (eV)		4.18	4.26
Dielectric Permittivity		10	100
Conduction band density of states (cm^−3^)		2.20 × 10^18^	2.20 × 10^18^
Valence band density of states (cm^−3^)		1.80 × 10^19^	1.80 × 10^19^
Electron (cm^2^/Vs)		1 × 10^2^	2
Hole Mobility (cm^2^/Vs)		2.5 × 10^1^	2
Shallow uniform donor density N_D_ (cm^−3^)		1 × 10^18^	0
Shallow uniform acceptor density N_A_ (cm^−3^)		0	

**Fig. 7:**
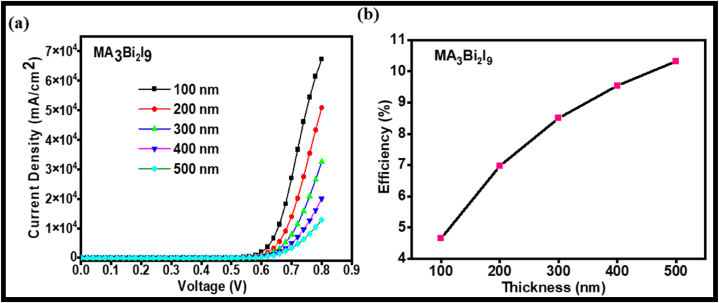
(a) J-V curves, and (b) Efficiency plot for MA_3_Bi_2_I_9_ for various thicknesses of the absorber layer.

It was observed that efficiency increases with an increase in the thickness of the absorber layer from 100 to 500 nm (Table 4a, Table 4b, Table 4c). Here, the MA_3_Bi_2_I_9_ halide perovskite simulated using a p-n junction device with perovskite acting as p-layer and CdS [66] acting as n-layer along with Au and Al as the top & bottom contact, respectively, in the solar device structure as shown in Fig. 6 [67]. Due to the unavailability of simulation input parameters of n- & p-layer of FA- & MA-based halide perovskites, we have studied all the P–V parameters of MA_3_Bi_2_I_9_ perovskite with thickness variation. Fig. 7 shows the J-V curve and efficiency plot of MA_3_Bi_2_I_9_ perovskite for thickness variation from 100 to 500 nm. Here, Fig. 7(b) showing the thickness variation of MA_3_Bi_2_I_9_ vs efficiency confers that an increase in thickness of perovskite film increases the efficiency signifying the film with a thickness of 500 nm can be optimized for optoelectronic devices with enhanced efficiency.

## 5 Conclusion

This work reports the development of FA-based Pb-free Bi-mixed halide (FA_3_Bi_2_I_x_Cl_1-x_ and FA_3_Bi_2_Br_x_Cl_1-x_) and MA-based Bi-pure halide (MA_3_Bi_2_X_9_ (X = Cl/Br/I)) perovskite materials using LARP technique. Here, we studied that structural modification with change in A-organic cation greatly impacts the structural, optical and optoelectronic properties of the A_3_B_2_X_9_ perovskite structures. These perovskite NCs were characterized using XRD, UV–Vis and PL techniques to determine the structural and optical properties of the perovskite mentioned above materials. Due to bismuth incorporation, the XRD pattern and UV–vis spectra have confirmed the improved moisture stability for these halide perovskite samples. This paper reports that the efficiency of the MA_3_Bi_2_I_9_ perovskite absorber layer increases with respect to the thickness variation from 100 to 500 nm. Thus, the PCE of MA-based Pb-free halide perovskites must be enhanced using post-synthesis chemical techniques with thickness variation to utilize for commercial applications. Therefore, this work highlights that substituting A-organic cations based on their ionic radius increases band-gap and octahedral deformation in Bi-halide perovskite-based devices. It also focuses on the importance of halide ions in maintaining the charge neutrality between cations & anions. At the same time, mixed halide perovskites are also known as a potential candidate in future Pb-free organic-inorganic perovskite-based devices. Thus, it concludes that the non-toxic Bi-halide-based perovskite NCs can be recognized as a probable contender for optoelectronic applications.

## Data availability Statement

Data will be made available on request.

## CRediT authorship contribution statement

**Sonali Mehra:** Conceptualization, Data curation, Investigation, Methodology, Writing – original draft. **Mamta:** Data curation, Resources, Software, Writing – original draft. **V.N. Singh:** Resources, Software, Writing – review & editing. **Govind Gupta:** Resources, Software. **A.K. Srivastava:** Supervision, Writing – review & editing. **Shailesh Narain Sharma:** Supervision, Validation, Visualization, Writing – review & editing.

## Declaration of competing interest

The authors declare that they have no competing financial interest or personal relationships that could have appeared to influence the work reported in this paper.
